# HbD Punjab/HbQ India Compound Heterozygosity: An Unusual Association

**DOI:** 10.4084/MJHID.2014.072

**Published:** 2014-11-01

**Authors:** Stacy Colaco, Reema Surve, Pratibha Sawant, Anita Nadkarni, Kanjaksha Ghosh, Roshan Colah

**Affiliations:** National Institute of Immunohaematology, Indian Council of Medical Research, 13^th^ Floor, New Multistoried Building, King Edward Memorial Hospital Campus, Parel, Mumbai – 4000 12.

## Abstract

**Background:**

Haemoglobinopathies are the commonest hereditary disorders in India and pose a major health problem. Both beta thalassaemia and structural haemoglobin variants are relatively common in northwestern India. Here we report a 29-year-old Sindhi female who was referred to us for a haemoglobinopathy work up and genetic counseling since her spouse was a classical beta thalassaemia carrier.

**Method:**

A complete blood count was done on an automated cell counter. Haemoglobin analysis was carried out using HPLC Variant Haemoglobin Testing System. The cellulose acetate electrophoresis was carried out [pH 8.9]. Confirmation of mutations was done by automated DNA sequencing.

**Results:**

HPLC analysis showed four major peaks, HbA_0_, a peak in the HbD window, an unknown peak [retention time 4.74 minutes] and a peak in the HbC window. The HbA_2_ level was 2.2%, and the HbF level was 0.7%. Cellulose acetate electrophoresis at alkaline pH, a slow moving band was seen at the HbS/D position along with a prominent band at the HbA_2_ position. DNA sequencing of the β and α genes showed presence of the two hemoglobin variants: Hb D [β 121GAA → CAA] and Hb Q [α 64 AAG → GAG]. The δ globin gene was normal. The additional peak in the HbC window was due to the formation of a heterodimer hybrid.

**Conclusion:**

Both HbD Punjab and HbQ India are relatively common in India, but their co-inheritance has not been described in the country. This case is the third report of compound heterozygosity for HbQ India/HbD Punjab haemoglobinopathy globally and the second one from India.

## Introduction

Inherited abnormalities of haemoglobin synthesis include a myriad of disorders ranging from the thalassaemia syndromes to structurally abnormal haemoglobin variants. Identification of these abnormalities is immensely important epidemiologically and aid in the prevention of more severe haemoglobin disorders.^1^ Haemoglobinopathies resulting from mutations in the α or β globin gene clusters are the most common inherited disorders in humans. Single nucleotide substitutions can lead to amino acid replacements that cause haemolytic anaemias, such as sickle cell disease, or haemoglobins that are unstable or have altered oxygen affinity.^2^ Molecular defects in either regulatory or coding regions of the human α, β or δ globin genes can minimally or drastically reduce their expression, leading to α, β or δ thalassaemia.^3^ Other sequence changes have little or no effect on haemoglobin function, but are useful polymorphisms for genetic studies. About 7% of the world population carries a globin gene mutation, and in the vast majority of cases it is inherited as an autosomal recessive trait.^1^ To date, over 1,200 different mutant alleles have been characterized at the molecular level^4^ and each country has its own spectrum of Hb variants and thalassemia mutations.^2^ Here, we describe a very rare compound heterozygous haemoglobinopathy, HbD Punjab/HbQ India [αα^Q India^ ββ^D Punjab^], which has been reported only two times in the literature to date, to the best of our knowledge.^5^,^6^

## Materials and methods

A 29-year-old female belonging to the Sindhi community, originating from the Sindh province of Pakistan was referred to us for screening for haemoglobinopathy since her husband was a classical β thalassaemia trait. After obtaining an informed consent, 5 mL of intravenous blood was collected. A complete blood count was done on an automated haematology analyzer [Sysmex K-1000, Kobe, Japan]. Reticulocyte staining was performed using New Methylene Blue dye. Sickling test was done using a solution of 2% sodium metabisulphite to differentiate between Hb S and Hb D.^7^ Haemoglobin analysis was done by HPLC on the Variant Haemoglobin Testing System [Classic] [Bio-Rad Inc, CA, USA]. Cellulose acetate electrophoresis was carried out at [pH 8.9].^6^ The eight common α thalassaemia determinants in the Indian population were screened using Multiplex PCR.^8^ The molecular characterization of α, β and δ gene was done using automated DNA sequencing [Applied BioSystems, Foster City, CA, USA].

## Results

**Figure 1:**
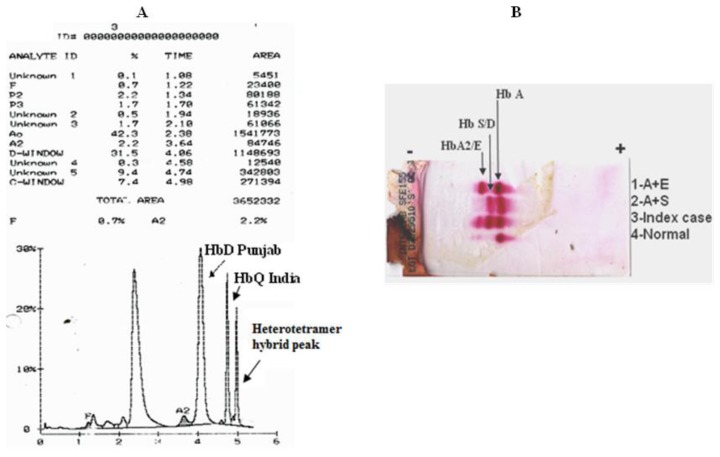
Haemoglobin analyses by [A] HPLC and [B] Electrophoresis at alkaline pH

The hematological analysis of the patient showed a marginal decrease in the haemoglobin level with normal red cell indices (Table 1). Reticulocyte count was 1.5%. HPLC analysis, using the β thalassaemia short program, showed a peak of 31.5% with a retention time of 4.06 minutes in the Hb D window, followed by an unknown peak of 9.4% with a retention time of 4.74 minutes as well as a peak eluting in the Hb C window [7.4%] with a retention time of 4.98 minutes. The unknown peak at 4.74 minutes was suggestive of Hb Q India (Fig 1A). Cellulose acetate electrophoresis at alkaline pH [8.9] showed three bands viz Hb A, a band at the S/D position and Hb A2 (Fig 1B). Confirmation of absence of HbS was done by sickling test that was negative. α globin gene analysis showed the absence of the common α thalassaemia determinants and absence of α globin gene triplication. Direct DNA Sequencing of the α globin gene revealed the presence of HbQ India [α 64Asp → His] and sequencing of the β globin gene showed the presence of HbD Punjab [β 121Glu → Gln]. The δ globin genes showed absence of any mutation.

## Discussion

Genomic research has rapidly evolved during the past decades, and the scientific approach to genetic-based understanding of human disease has changed along with technological development. This led to the discovery of a large list of Hb variants. Here we report a very rare and unusual case of double heterozygosity of the α chain variant HbQ India with the non-α chain variant HbD Punjab. The relative electrophoretic mobility of HbD Punjab and HbQ India are practically indistinguishable during routine screening on alkaline electrophoresis.^9^

Higgins et al.,^5^ reported in 2012 the first case of a HbD Punjab/HbQ India compound heterozygosity in female of Indian origin. Her investigation for haemoglobinopathies showed four major peaks Hb A, HbD Punjab, HbQ India and HbD Punjab/HbQ India. The heterotetramer hybrid peak eluting in the HbC window reported by them was also seen in our case. In electrophoresis at alkaline pH, Higgins et al.^5^ observed bands at the C [HbD Punjab/HbQ India], S [HbD] and A [HbA] positions, while the electrophoresis at acid pH showed two bands, at the S position [HbD Punjab/HbQ India] and at the A position [HbA and HbD Punjab]. Similar observations were reported in two Indian patients double heterozygous for HbQInida/HbD Punjab by Mutreja et al. 2013.^6^

It has been suggested that of the HbE and HbD mutations were originated in India and dispersed to other parts of the world due to migration.^10^ HbQ, a rare α chain variant, was first reported by Vella et al., in 1958 in a Chinese family.^11^ Three variants of HbQ have been described, namely, HbQ-India, HbQ-Thailand [c.223G>C] and HbQ-Iran [c.226G>C].^2^ The first case of HbQ India was reported by Sukumaran et al., in 1972 in a Sindhi family with associated β thalassaemia.^12^ The affected residue, α64 [E13], lies on the surface of the haemoglobin tetramer and charge changes at this position do not alter the tertiary structure of the haemoglobin molecule.^13^ Hence, the presence of HbQ India does not impart any functional deficit and lacks any clinical manifestation.^14^

64 cases of HbQ India trait and its interaction with β thalassaemia have also been reported from India.^15^ However, a few concerns have been raised for considering it as an entirely benign disorder; Panigrahi et al.,^16^ in 2005 reported mild anaemia and microcytosis in heterozygotes of HbQ India and observed that apart from co-existent α globin/β globin genotypes, iron deficiency also affects HbQ levels.

Das et al.,^6^ in 2013 studied the intra-ethnic heterogeneity of the haemoglobinopathies in 1,498 Sindhis and observed the incidence of HbD Punjab and HbQ-India to be 1.87% and 0.4% respectively. Giordano et al.^17^ reported in 2006 a β thalassemia heterozygote where the diagnosis of high A2 β thalassemia was masked by the presence of an α chain variant, HbJ Meerut. The presence of α chain variants leads to the generation of split HbA_2_ peaks thereby reducing the HbA_2_ value by 20%. The generation of this variant might be the reason that HbA_2_ level in our case (2.2%) was lower than usual.

Rahimi et al.^18^ reported in 2007 an unusual case of an Iranian child with HbQ-Iran [alpha75 (EF4) Asp → His]/- α 3.7 kb/βIVSII.1 G → A, who presented as a minor beta-thalassemia with moderate anemia. In our case as the husband of the propositus was a β thalassemia carrier, the couple was at risk of having a child with double heterozygosity for HbD Punjab - β thalassemia, or HbQ-India - β thalassemia or HbQ-India -Hb D Punjab - β thalassemia. A combination of these hemoglobinopathies in our experience does not lead to a severe disorder. Hence, the couple was not advised to undergo prenatal diagnosis. However, when one of the partners has a β thalassemia trait, it is always advisable to do the full molecular work up of the spouse to ensure that they are not at risk of having a child with a severe hemoglobin disorder.

## Conclusion

Often unusual clinical presentations may be explained by the interaction of several Hb abnormalities and their identification may require further investigations. This is the third literature report of the compound heterozygosity of HbQ India/HbD Punjab. The couple was genetically counseled and was advised not to undergo prenatal diagnosis.

## Figures and Tables

**Table 1:** Hematological analysis of the patient

Parameters	Case
Age/Sex	29/F
WBC × 10^3^/μl	9.4
RBC × 10^6^/μl	4.25
Hb g/l	119
HCT %	33.9
MCV fl	79.8
MCH pg	28
MCHC g/l	351
RDW %	13.5
Hb A_2_ %	2.2
Hb F %	0.7
Hb D window	31.5
Unknown peak [RT 4.74 mins]	9.4
Hb C window	7.4
